# Beyond dopamine: exploring endocannabinoids in Parkinson’s disease

**DOI:** 10.20517/and.2024.18

**Published:** 2024-08-06

**Authors:** Disa Basu, Nannan Yang, Jinhui Ding, Lupeng Wang, Zhenhua Liu, Beisha Tang, Huaibin Cai

**Affiliations:** 1 Transgenics Section, Laboratory of Neurogenetics, National Institute on Aging, National Institutes of Health, Bethesda, MD 20892, USA.; 2 Department of Neurology, The First Affiliated Hospital of Zhengzhou University, Zhengzhou 450001, Henan, China.; 3 Computational Biology Group, Laboratory of Neurogenetics, National Institute on Aging, National Institutes of Health, Bethesda, MD 20892, USA.; 4 Department of Neurology, Xiangya Hospital, Central South University, Changsha 410008, Hunan, China.; 5 Centre for Medical Genetics and Hunan Key Laboratory of Medical Genetics, School of Life Sciences, Central South University, Changsha 410008, Hunan, China.; 6 National Clinical Research Center for Geriatric Disorders, Xiangya Hospital, Central South University, Changsha 410008, Hunan, China.; 7 Key Laboratory of Hunan Province in Neurodegenerative Disorders, Central South University, Changsha 410008, Hunan, China.

**Keywords:** Parkinson’s disease, dopamine, endocannabinoid, diacylglycerol lipase β, 2-arachidonoyl-glycerol, nigrostriatal dopaminergic neurons, cannabinoid receptor 1, monoacylglycerol lipase

## Abstract

Parkinson’s disease (PD) is a prevalent degenerative movement disorder largely attributed to the dysfunction of dopamine transmission in the basal ganglia. However, the role of the endocannabinoid (eCB) system (ECS) in PD pathology and symptomatology is often overlooked in discussions. Recent research, including our own, has identified multiple homozygous loss-of-function variants in diacylglycerol lipase β (DAGLB), an enzyme involved in the synthesis of 2-arachidonoyl-glycerol (2-AG) - the most abundant eCB in the brain - in individuals with early-onset autosomal recessive Parkinsonism. These genetic findings strongly link eCB deficiency with the etiopathogenesis of PD. Exploring the roles of DAGLB and 2-AG signaling in PD and dopamine transmission could provide a new perspective on PD treatments, focusing on the function of the ECS and the pathophysiological implications of its disruption.

## INTRODUCTION

Parkinson’s disease (PD) ranks as the second most common degenerative neurological disorder after Alzheimer’s disease^[1]^. As one of the fastest-growing neurological conditions^[2]^, PD affects millions of elderly people worldwide. Persons with PD display progressive motor symptoms, such as resting tremor, slowed movement, impaired posture and balance, and rigid muscles^[3]^. Additionally, they frequently experience non-motor symptoms including chronic pain, depression, dementia, sleep and autonomic dysfunction, and others^[4]^. While medications and surgical interventions can enhance motor performance in PD persons, treatments for non-motor symptoms remain limited^[5]^. Furthermore, long-term medication usage can lead to severe side effects like dyskinesia and impulse control disorders^[6,7]^. Therefore, there is an ongoing need for new mechanistic insights and therapeutic agents to improve the treatment outcomes for the growing number of PD persons^[8]^.

The nigrostriatal dopaminergic neurons (DANs) play a crucial role in regulating the vigor of movement^[9]^ and motor learning^[10]^. The activity of nigrostriatal DANs and dopamine release can be dynamically regulated by diverse presynaptic inputs, of which the dopamine receptor D1-expressing direct pathway spiny projection neurons (dSPNs) in dorsal striatum being the major inhibitory contributors^[10–14]^. The axon terminals of dSPNs are rich in cannabinoid receptor 1 (CB1R)^[15,16]^, which may interact with 2-arachidonoyl-glycerol (2-AG) and anandamide (AEA) released from the nigrostriatal DANs. As retrograde neuromodulators, eCBs suppress presynaptic neurotransmitter release through the G protein-coupled receptor (GPCR) CB1Rs and regulate a variety of physiological processes, such as motor learning, stress response, and memory^[17–21]^. Midbrain DANs can produce and release eCBs from soma and dendrites^[22]^. Both diacylglycerol lipase α (DAGLA) and its homolog diacylglycerol lipase β (DAGLB) mediate the biosynthesis of 2-AG in the brain^[23]^. While DAGLA catalyzes most of the 2-AG production in the brain^[24–26]^, DAGLB appears to be the main 2-AG synthase in nigrostriatal DANs^[27]^.

Confounding upregulation and downregulation of eCBs and receptors have been observed in individuals with PD and related animal models, including genetically modified mice containing PD-related α-synuclein and Parkin mutations, as well as neurotoxin-induced dopamine depletion in rodents and non-human primates, as extensively reviewed previously^[20,28,29]^. For example, individuals with PD had lower plasma and cerebrospinal fluid (CSF) levels of 2-AG and higher CSF levels of AEA compared to healthy controls^[30]^. Consistently, overexpression of α-synuclein in rodent DANs also led to a reduction in the 2-AG levels^[31]^. Therefore, the abnormal reduction of 2-AG levels in the plasma and CSF of individuals with PD could be used as biomarkers for the early detection of PD. However, whether these changes in eCB signaling contribute to the disease or represent compensatory responses remains debated.

Recently, multiple homozygous loss-of-function variants in the *DAGLB* gene have been associated with early-onset autosomal recessive Parkinsonism^[27,32]^. These genetic findings provide compelling evidence supporting the role of eCB deficiency in PD pathogenesis. Understanding how the eCB system modulates dopaminergic transmission in motor control and safeguards against DAN degeneration could offer novel insights into the pathogenic mechanisms and treatment strategies for PD.

## ENDOCANNABINOID SYSTEM IN THE BRAIN

**Figure 1.**
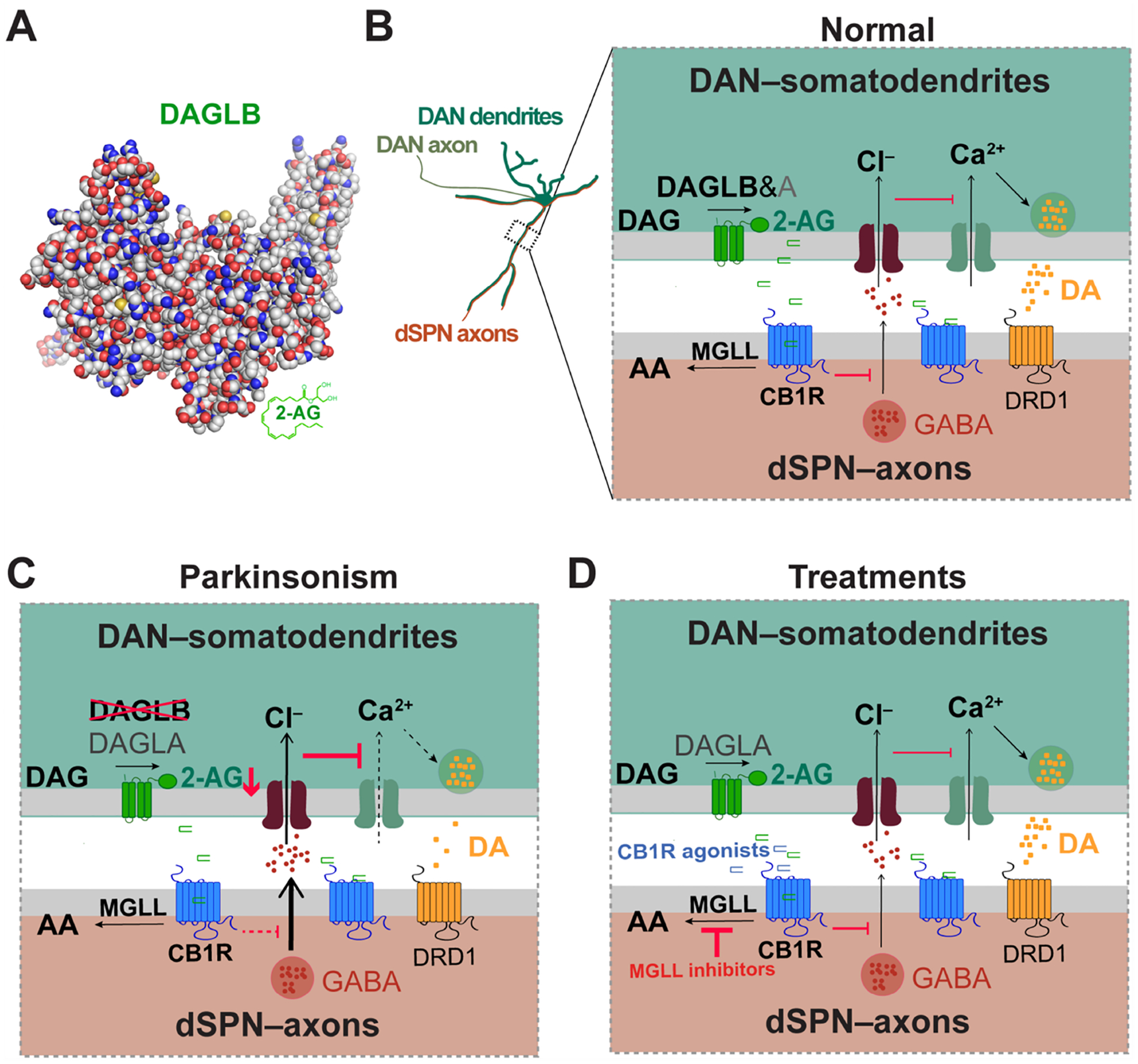
DAGLB and eCB signaling in DANs. (A) The cartoon illustrates the 3D protein structure of DAGLB predicted by AlphaFold; (B) DAGLB is suggested to be the main synthase of the eCB 2-AG in DANs^[27]^. The 2-AG produced in the postsynaptic DANs by DAGLB and DAGLA are released into the synaptic cleft upon neuronal activation. They then retrogradely bind to CB1R in the presynaptic sites of DRD1-expressing dSPNs, leading to the suppression of neurotransmitter GABA release. GABA suppresses the activity of DANs and DA release. MGLL, associated with the presynaptic sites, mediates the degradation of 2-AG into AA and glycerol, thereby terminating the 2-AG-mediated presynaptic inhibition; (C) The loss-of-function variants in the *DAGLB* gene have been associated with early-onset recessive Parkinsonism, resulting in a reduction of 2-AG production from DANs, increased inhibitory inputs from dSPNs, and reduced dopamine release; (D) Augmentation of 2-AG levels in nigral regions, either through the supplementation of CB1R agonists or the inhibition of MGLL hydrolysis activity, could restore local 2-AG levels and dopamine transmission, offering a potential treatment avenue for the disease. DAGLB: Diacylglycerol lipase β; eCB: endocannabinoid; DANs: nigrostriatal dopaminergic neurons; 2-AG: 2-arachidonoyl-glycerol; DAGLA: diacylglycerol lipase α; CB1R: cannabinoid receptor 1; DRD1: dopamine receptor D1; dSPNs: direct pathway spiny projection neurons; GABA: gamma-aminobutyric acid; DA: dopamine; MGLL: monoacylglycerol lipase; AA: arachidonic acid.

The endocannabinoid (eCB) system (ECS), which includes eCBs, their receptors, and synthetic and degradation enzymes, stands as one of the most important neuromodulatory systems in the mammalian brain [Figure 1]. The eCBs represent a distinct class of lipid signaling molecules widely distributed in the human body^[33,34]^. The primary eCB in the central nervous system (CNS) is 2-AG. 2-AG is synthesized from 1-acyl-2-arachidonoyl-sn-glycerol [diacylglycerol (DAG)] through hydrolysis by DAGLA and DAGLB^[35]^. The degradation of 2-AG mainly occurs through the hydrolysis by monoacylglycerol lipase (MGLL) to arachidonic acid (AA) and glycerol. A small portion is degraded by α/β-hydrolase 6 and α/β-hydrolase 12^[36]^. The physiological effects of 2-AG are primarily mediated by CB1R and cannabinoid receptor 2 (CB2R)^[37,38]^. CB1Rs are abundant in the CNS, especially in the basal ganglia, cortex, and hippocampus, predominantly distributed in axon terminals^[39,40]^. CB2Rs are expressed at very low levels in the brain, limited to specific neurons, and are abundant in activated microglia and astrocytes^[41,42]^.

Unlike conventional neurotransmitters and modulators, 2-AG acts as a retrograde messenger and can provide feedback regulation of neurotransmitter release from presynaptic neurons [Figure 1]^[43]^. The production and release of 2-AG are distinctively different from the well-studied amino acid neurotransmitters, such as glutamate and dopamine. Instead of being synthesized, stored, and released, 2-AG is produced and released “on demand”. DAGLs are activated to synthesize 2-AG upon the activation of postsynaptic neurons. Following release from the postsynaptic neurons, 2-AG diffuses across the synaptic cleft and binds to CB1Rs on the presynaptic sites. A recent study highlights the involvement of synuclein family proteins in regulating the transport and postsynaptic release of 2-AG during synaptic transmission^[44]^. However, further research is required to gain a comprehensive understanding of the molecular mechanisms governing the production, transport, and release of 2-AG and other eCBs in the brain.

When CB1Rs on the presynaptic membrane are activated, they inhibit the activity of adenylate cyclase via the inhibitory GPCR-mediated intracellular signaling transduction, resulting in reduced neurotransmitter release from the presynaptic terminals^[45]^. This regulation exists in various brain regions, such as the cortex, hippocampus, basal ganglia, and cerebellum, participating in synaptic plasticity, regulating learning and memory, drug addiction, and motor learning. Behavioral studies in CB1R knockout mice show that CB1Rs are crucially involved in various forms of learning, including spatial learning^[46]^, fear regulation^[47]^, eyeblink conditioning^[48]^, and habit formation^[49,50]^ through modulating the synaptic transmission and plasticity in hippocampus, amygdala, cerebellum, and basal ganglia.

## ECS IN BASAL GANGLIA

In the dorsal striatum, CB1Rs are highly expressed in both dSPN and dopamine receptor D2-expressing indirect pathway projection neurons (iSPNs)^[51]^. These neurons project to the substantia nigra (SN) and globus pallidus in basal ganglia. The axon terminals of dSPNs, particularly those from the patch (also known as striosome) compartments, form a distinctive striosome-dendron bouquet structure^[16]^. This structure interacts with the dendrites of DANs that extend into the SNr region^[13,16]^. Within these striosome-dendron bouquets, CB1Rs are notably enriched in the axon terminals of dSPNs^[15]^. Moreover, genetic deletion of CB1R in dSPNs affects the synaptic formation with DANs during development^[52]^, suggesting a critical involvement of eCB signaling in the establishment of neural circuitry.

*Ex vivo* brain slice electrophysiological recordings have found that after depolarization of DANs in the substantia nigra pars compacta (SNc), inhibitory postsynaptic currents decrease, leading to depolarization-induced suppression of inhibition (DSI)^[53]^. When given a CB1R antagonist AM251, this DSI effect disappears. It is possible that after depolarization of DANs, 2-AG is released to the presynaptic membrane, activating CB1Rs, which reduces gamma-aminobutyric acid (GABA) released by dSPNs, resulting in DSI. When given a CB1R antagonist, 2-AG cannot activate CB1Rs, and DSI is disrupted^[53]^. Additionally, *in vivo* experiments in mice have shown that intraperitoneal administration of a CB1R agonist can increase the firing frequency of DANs in the SNc and ventral tegmental area (VTA)^[54,55]^, as well as dopamine release^[55,56]^. Moreover, in brain slice experiments, local application of a CB1R agonist can increase the single spike firing and burst rate of DANs^[57,58]^, indicating that CB1Rs can regulate the excitability of DANs by modulating presynaptic inputs in the midbrain.

## DAGLA AND DAGLB IN THE BRAIN

While both DAGLA and DAGLB contribute to the production of 2-AG, the specific functions of these two enzymes in different cell populations under varying conditions remain an active area of research. In neurons, DAGLA protein is predominantly localized to the postsynaptic sites of dendritic spines, likely due to the interaction between its C-terminal PPxxF domain and the postsynaptic density protein Homer^[24,25,59]^. The deletion of the Homer-binding domain does not impact the enzymatic activity of DAGLA but disrupts its cell surface and postsynaptic targeting^[59]^. Furthermore, genetic variants in the *DAGLA* gene that lead to the truncation of the C-terminal Homer-binding domain have been associated with neuro-ocular abnormalities in affected children^[60]^. This underscores the importance of proper subcellular targeting for the normal functioning of DAGLA. The DAGLB protein does not possess the homer-binding domain in its C-terminal, and its localization at the postsynaptic site remains to be elucidated. The absence of specific antibodies for tissue staining complicates this investigation. However, given that the CB1R-containing axon terminals are in close proximity to the dendrites and cell bodies of postsynaptic neurons^[15]^ and considering that 2-AG functions within a limited range (~10 mm) from the release sites^[61,62]^, it is plausible that DAGLB is distributed near postsynaptic sites for localized 2-AG production and release. Alternatively, DAGLB might produce 2-AG intracellularly in yet-to-be-identified organelles. The 2-AG could then act on conventional or unconventional receptors within cells to regulate the functions of mitochondria or other subcellular structures^[63,64]^. However, how the enzymatic activity of DAGLB is regulated is largely unexplored and merits further investigation.

Studies featuring *Dagla* and *Daglb* knockout mice have been critical in deciphering which of these two enzymes serves as the main 2-AG synthase across various tissue and cell types. These investigations consistently identify DAGLA as the predominant 2-AG synthase of the brain. Specifically, *Dagla* knockout mice have been shown to exhibit an 80%-90% reduction in brain 2-AG levels^[24]^. Additionally, these studies reveal that 2-AG-mediated retrograde feedback inhibition is completely abolished in the cerebellum, hippocampus, and striatum of *Dagla* knockout mice, as well as stimulus-induced 2-AG production^[25]^. In contrast, *Daglb* knockout mice do not show significant differences from control mice in these aspects. Therefore, these early studies do not support a critical role for DAGLB-mediated 2-AG synthesis during synaptic transmission. However, it is worth noting that DAGLB has been shown to regulate axonal outgrowth during neuronal development^[65]^.

## DAGLA AND DAGLB IN THE SN AND PARKINSONISM

**Figure 2.**
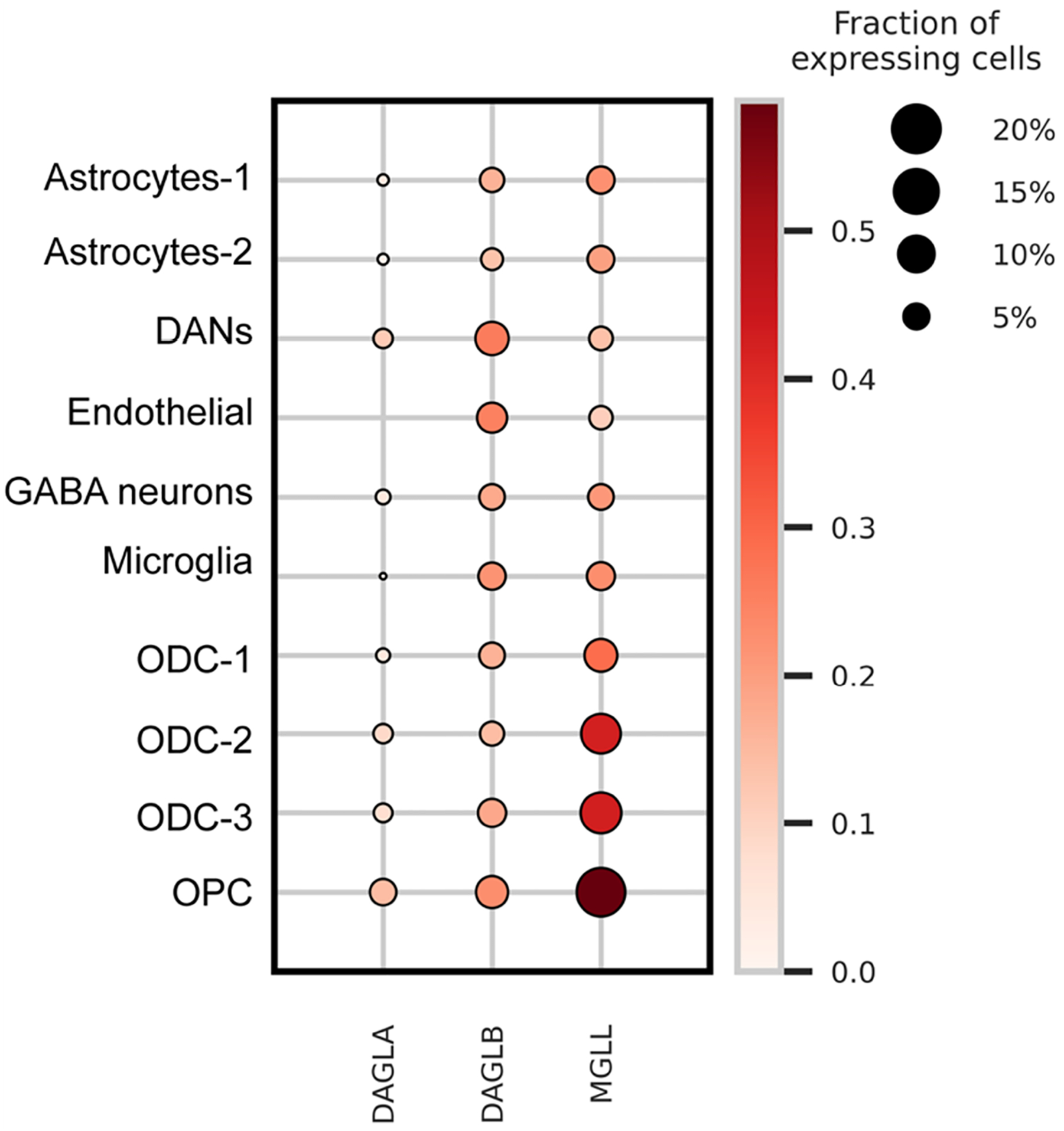
Expression of DAGLA, DAGLB, and MGLL in human SN region. Dot plot of a previously published single-nuclei transcriptomic dataset from the human SN region^[66]^. It compares the gene expression levels of *DAGLA*, *DAGLB*, and *MGLL* in type 1 and type 2 astrocytes, DANs, endothelial cells, GABAergic neurons, microglia, and type 1–3 oligodendrocytes, as well as OPCs. The size of each circle indicates the percentage of cells expressing a given gene within the cell population. The color intensity, ranging from light to dark red, represents the increase in gene expression level. DAGLA: Diacylglycerol lipase α; DAGLB: diacylglycerol lipase β; MGLL: monoacylglycerol lipase; SN: substantia nigra; DANs: nigrostriatal dopaminergic neurons; GABA: gamma-aminobutyric acid; OPCs: oligodendrocyte precursor cells.

Although DAGLA is commonly recognized as the main 2-AG synthase in the CNS, both bulk and single-nuclei transcriptomic analyses have revealed that *DAGLB* mRNA is more abundant than *DAGLA* in nigrostriatal DANs and other cell types within the SN^[27,66,67]^ [Figure 2]. Furthermore, genetic deletion of *Daglb* selectively in mouse nigrostriatal DANs markedly reduces the production of 2-AG in the SN^[27]^, supporting the notion that DAGLB is not only enriched in the DANs but also functionally serves as the primary 2-AG synthase in SN.

The expression and functional significance of DAGLB in nigrostriatal DANs may help to explain why multiple loss-of-function variants in DAGLB have been associated with early-onset Parkinsonism^[27,32]^. These genetic variants either disrupt *DAGLB* transcription or impair DAGLB protein stability^[27]^, resulting in a complete loss of DAGLB function. It is plausible to suggest that these Parkinsonism-associated *DAGLB* variants could lead to decreased 2-AG production by nigrostriatal DANs, providing a previously unrecognized pathogenic mechanism for the disease.

This proposed pathogenic mechanism focuses on the reduced activity of nigrostriatal DANs due to the loss of 2-AG-mediated disinhibition of the dSPNs-induced inhibitory inputs to the DANs^[27]^. The dSPNs exert inhibitory GABAergic inputs onto the nigrostriatal DANs, suppressing their activity and dopamine release^[10,11,13]^. The axon terminals of the dSPNs are rich in CB1R expression and are susceptible to binding by 2-AG produced and released from the postsynaptic sites of nigrostriatal DANs, which provide negative feedback to reduce the dSPN activity while enhancing the activity of nigrostriatal DANs^[15,27]^.

In the context of DAGLB dysfunction, impaired 2-AG synthesis in nigrostriatal DANs would prevent this disinhibition, potentially contributing to the development of PD-like symptoms. Conversely, augmentation of 2-AG levels by pharmacologically inhibiting its hydrolysis boosts DAN activity and dopamine release, thereby alleviating motor abnormalities^[27]^. Given the genetic and pathophysiological evidence suggesting that deficiency in DAGLB-mediated 2-AG production contributes to the etiopathogenesis in PD, enhancing 2-AG transmission within the SN may present a viable therapeutic approach for the disease.

## ANIMAL MODELS FOR STUDYING THE ECS IN THE BASAL GANGLIA AND PD

While there are a limited number of animal models currently available for exploring the role of eCB signaling in PD pathogenesis, numerous models have been established to investigate the roles of various ECS components in different basal ganglia neuronal populations. For example, the genetic deletion of CB1R specifically in mouse dSPNs resulted in motor impairments like those observed in mice with selective deletion of the *Daglb* gene in adult nigrostriatal DANs^[27]^. This finding supports the notion that DAGLB-mediated 2-AG signaling between nigrostriatal DANs and dSPNs plays an important role in motor function, especially the retention of newly acquired motor skills^[27]^. However, *Daglb* germline knockout mice, which lack functional DAGLB from the embryonic stage and mimic the loss-of-function genetic variants in PD persons, do not display any noticeable motor phenotypes^[24,26,27,35]^. This discrepancy highlights the need for further exploration into the roles of DAGLB and 2-AG in motor control, learning, and PD pathogenesis.

In addition to the role of DAGLB-dependent 2-AG production in nigrostriatal DANs and its implication in PD pathogenesis, there are intriguing insights into the interplay between DAGLA-mediated 2-AG and dopamine within the basal ganglia, and their influence on specific behaviors. Targeted knockdown of *Dagla* gene expression in mouse dSPNs leads to reduced 2-AG levels in the striatum. This results in the loss of 2-AG-mediated retrograde feedback inhibition, leading to an increase in glutamatergic release onto the dSPNs^[68]^. Such alterations in 2-AG signaling prompt mice to exhibit autistic-like behaviors, including excessive self-grooming, reduced exploratory drive, and impaired sociability^[68]^. In *Dagla* conditional knockout mice (cKO) targeting dSPNs, the reduction of 2-AG produced in the dSPNs blunts the sedative effects of ethanol observed in rats. This suggests that 2-AG signals are important for these sedative effects when they are present at normal levels^[69]^. Moreover, viral vector-mediated knockdown of *Dagla* in mouse VTA DANs reveals the role of DAGLA-produced 2-AG as an integral part in the acquisition and invigoration of dopamine signals during conditioned reward-seeking behavior^[70]^. The *Dagla*-viral knockdown mice were significantly less motivated to pursue high-effort, cue-driven rewards compared to the controls^[70]^. It is apparent that the ability of 2-AG to facilitate the depolarization suppression of excitation and inhibition plays a pivotal role in multiple signaling pathways within the basal ganglia^[45]^. Considering that the relevance of 2-AG signaling in movement control and PD pathogenesis is a relatively recent discovery^[27]^, there is still much to be elucidated.

## ECS-BASED TREATMENTS IN PRECLINIC PD ANIMAL MODELS

PD treatments that target ECS are relatively limited. Understanding the current state of these therapies can shed light on how DAGLB might enhance these existing methods. Notably, research has explored the potential of another eCB ligand, AEA, for PD treatment. Results have been mixed: while inhibiting AEA hydrolysis in mouse PD models showed no effect on the nigrostriatal pathway or respiratory deficits^[71,72]^, it preserved motor function^[71]^. However, given our focus on 2-AG, we will concentrate on therapies that are based on modulating 2-AG levels. Many therapies accomplish this through the manipulation of MGLL [Table 1].

MGLL accounts for 85% of 2-AG hydrolysis in the brain and acts as a limiting factor in 2-AG signaling pathways^[73,74]^. This MGLL-dependent 2-AG hydrolysis serves two primary functions: eCB signaling and neuroinflammation. In terms of eCB signaling, it is important to note that MGLL and the DAGLs are distinct in their expression patterns and subcellular localizations. While MGLL is mainly restricted to presynaptic sites, DAGLs often reside in the postsynaptic density^[26,73]^. Upon depolarization, postsynaptic DAGLs synthesize 2-AG until it reaches the levels required for 2-AG-mediated synaptic transmission and plasticity. Since MGLL is confined to the presynaptic sites, it does not interfere with this postsynaptic elevation of 2-AG levels. However, MGLL can rapidly hydrolyze 2-AG that arrives at the presynaptic sites, enhancing the temporal specificity of the eCB signals.

MGLL is commonly characterized by its role in neuroinflammatory pathways. It hydrolyzes 2-AG into AA, a precursor for proinflammatory prostaglandins and eicosanoids implicated in the degeneration of nigrostriatal DANs associated with canonical PD neuropathology. That metabolic linkage between MGLL and proinflammatory prostaglandins forms the basis for discussions regarding MGLL as a potential target for innovative PD treatments. For instance, in 1-methyl-4-phenyl-1,2,3,6-tetrahydropyridine (MPTP) mouse models of PD, treatment with MGLL inhibitors such as JZL-184 and URB602 has been shown to offer neuroprotective effects by preventing the MPTP-induced degeneration of dopaminergic neurons in the midbrain^[75,76]^. This protective effect was also observed in *Mgll* knockout mice^[77]^. Likewise, the 6-hydroxydopamine (6-OHDA) PD model showed a reduced level of nigrostriatal DAN cell death when treated with MGLL inhibitor KML-29^[78]^. Furthermore, targeting MGLL for neuroprotection has shown behavioral benefits; MPTP mice treated with JZL-184 displayed improvements in Parkinsonian motor impairments^[75]^.

Preventing 2-AG hydrolysis undoubtedly elevates overall 2-AG levels throughout the brain^[27]^. Consequently, it is pertinent to inquire whether this increased 2-AG contributes to the anti-inflammatory effects observed when targeting MGLL. Lipopolysaccharide (LPS)-treated mice demonstrate an increase in brain eicosanoids and prostaglandins that promote inflammation, an increase that was predictably attenuated in both JZL-184-treated and *Mgll* knockout mice^[77]^. Disruption to 2-AG signaling, such as using 2-AG antagonists or genetic inactivating CB1R and CB2R receptors, had minimal effect on reducing the levels of proinflammatory agents^[77]^. Thus, the neuroprotective effects of MGLL inactivation appear to be independent of 2-AG elevation. These effects are more accurately attributed to the reduction in AA and the consequent decrease in proinflammatory prostaglandins, rather than any enhancement of 2-AG signaling pathways.

While 2-AG may not be actively involved in the neuroprotective effects of MGLL inactivation, our research indicates that 2-AG-mediated signaling within the striatonigral pathway is integral to motor function^[27]^. We demonstrated that viral knockdown of the *Daglb* gene in nigrostriatal DANs in mice led to reduced 2-AG release from the SN and deficits in motor skill learning. Subsequently, we administered JZL-184 to these mice, anticipating that it would inhibit 2-AG degradation in the SN and alleviate PD-like symptoms. Remarkably, JZL-184 treatment restored nigral dopamine release to levels comparable to control mice and reversed the observed impairments in motor skill learning^[27]^. In this context, our primary focus is on the impaired ability of DAGLB to synthesize 2-AG, compromising 2-AG signaling pathways essential for regulating nigrostriatal DAN activity through presynaptic inhibition of GABAergic inputs. Inhibiting MGLL with JZL-184 elevates 2-AG levels, contributing to the restoration of SN dopamine release and the recovery of motor skill learning. Therefore, gaining a deeper understanding of DAGLB and its role in regulating nigrostriatal DAN activity and dopamine release could pave the way for novel cannabinoid-based therapies for PD, aiming to restore lost 2-AG signaling pathways to alleviate its symptoms.

It is worth noting that the beneficial effects observed following JZL-184 administration are primarily attributed to the improvement of nigrostriatal DAN activity, since there was no degeneration of DANs in the *Daglb*-deficient mice^[27]^. While therapies aimed at restoring lost 2-AG signaling pathways may not be as effective in PD persons with extensive nigrostriatal DAN degeneration, they still hold promise for alleviating symptoms in persons with disease-causing *DAGLB* variants. Additionally, we speculate that these therapies have the potential to enhance 2-AG signals in the remaining nigrostriatal DANs.

Beyond inhibiting the hydrolysis of 2-AG, various eCB-like compounds and specific CB1R and CBR2 agonists have been explored for their potential beneficial effects in alleviating PD-related motor and non-motor symptoms in clinical trials and preclinical models^[79–81]^. These efforts have produced mixed results. However, an exploratory clinical trial of eCB-like cannabidiol seems to improve the mobility and mental states of individuals with PD^[82]^.

## CONCLUSIONS AND FUTURE PERSPECTIVES

In conclusion, recent advancements in identifying genetic variants in *DAGLB* causally associated with early-onset autosomal recessive Parkinsonism have illuminated the role of *DAGLB*-mediated 2-AG biosynthesis in the etiopathogenesis of PD. Compared to the studies on DAGLA, the normal physiological functions of DAGLB in neurons remain less explored. The limited study of DAGLB may be due to its lower expression levels in many neuronal populations compared to DAGLA. However, the increasing availability of single-cell transcriptomic profiling has revealed distinct neuronal subpopulations and non-neuronal cell types in the brain with higher levels of DAGLB expression than DAGLA, including nigrostriatal DANs [Figure 2]. This could help explain how *DAGLB* deficiency contributes to PD-related motor symptoms. Enhancing the production of 2-AG by nigrostriatal DANs could serve as a potential mechanistic-based therapeutic intervention to boost dopamine release and neuronal activity in persons with preserved nigrostriatal DANs. Indeed, an exploratory clinical trial of an eCB-like cannabidiol has shown promise in improving the mobility and mental states of persons with PD^[82]^.

DAGLA, although a minor 2-AG synthase in nigrostriatal DANs, may also contribute to the 2-AG production in *Daglb*-deficient DANs. Genetic deletion of both *Dagla* and *Daglb* in nigrostriatal DANs may provide a critical means to evaluate the pathophysiological role of DAN-derived 2-AG in motor and non-motor behaviors in PD. Additionally, since 2-AG is implicated in inflammation^[83]^, it is important to further investigate the roles of *DAGLB* in microglia, oligodendrocytes, and astrocytes by selectively deleting *Daglb* in these glial cells.

Beyond the brain, eCB signaling has been implicated in inflammatory responses in various parts of the body^[83]^. For example, 2-AG in myeloid promotes vascular inflammation and atherogenesis^[84]^. *Daglb* inactivation in mouse peritoneal macrophages attenuates lipopolysaccharide-induced release of proinflammatory cytokine tumor necrosis factor-a^[85]^. The underlying molecular and cellular mechanisms may be shared by the immune cells in the brain and provide clues to investigate potential neuroinflammatory reactions. However, given that the inhibition of DAGLB activity counteracts inflammatory responses^[85]^, we posit that *DAGLB* deficiency is less likely to directly induce the harmful neuroinflammation implicated in the pathogenesis of PD. Nonetheless, future studies will be needed to further elucidate the role of *DAGLB* and its regulation in microglia or other non-neuronal cells in PD.

Furthermore, recent clinical trials have suggested the potential benefits of cannabinoids in improving the non-motor symptoms of individuals with PD, including mood and sleep^[86–88]^. Notably, an increasing amount of evidence has indicated that the ECS is associated with the circadian system and sleep, with both ECS ligands and receptors showing diurnal variations and regulating the activity of suprachiasmatic nucleus during the sleep-wake cycle^[80]^. The underlying neural circuit mechanisms by which ECS modulation improves the mental state and sleep of individuals with PD are of interest for future research.

Compared to PD, there are much fewer studies on the involvement of ECS in other synucleinopathies such as multiple system atrophy (MSA) and dementia with Lewy body, as well as atypical PD, including progressive supranuclear palsy and corticobasal syndrome. A recent study indicates the involvement of ECS in MSA pathology^[89]^. However, more research is needed to document the changes in the ECS in these disorders.

## Figures and Tables

**Table 1.** List of ECS-based treatments in PD-related animal models

Model	What was administered to the model?	How did the treatment reverse PD-like symptoms in the model?
*Daglb* knockdown mouse	JZL-184 (MGLL inhibitor)	Rescued nigral dopamine release and motor skill learning^[27]^
MPTPmouse	JZL-184	Prevented nigrostriatal degeneration by curbing inflammatory responses in astroglia and microglia^[76–78]^. Preserved motor function as well^[78]^
MPTP mouse	URB602 (MGLL inhibitor)	Prevented MPTP-induced nigrostriatal degeneration^[76]^
MPTP mouse	DFU (COX-2 inhibitor)	Prevented MPTP-induced nigrostriatal degeneration. Led to an increase in 2-AG levels^[76]^
*Mgll* knockout mouse	LPS	Prevented LPS-induced DAN terminal loss and DA reductions in the SN and striatum^[77]^
6-OHDA mouse	KML-29 (MGLL inhibitor)	Prevented 6-OHDA-induced DAN cell death^[78]^
MPTP mouse	KML-29	Prevented MPTP-induced striatal dopamine depletion. Enhanced GDNF mRNA expression^[90]^
6-OHDA rat	D9-THC	Prevented 6-OHDA-induced DAN cell death^[91]^

ECS: Endocannabinoid system; PD: Parkinson’s disease; MGLL: monoacylglycerol lipase; MPTP: 1-methyl-4-phenyl-1,2,3,6-tetrahydropyridine; DFU: 5,5-dimethyl-3-(3-fluorophenyl)-4-(4-methylsulphonyl)phenyl-2-(5H)-furanone; 2-AG: 2-arachidonoyl-glycerol; LPS: lipopolysaccharide; DAN: nigrostriatal dopaminergic neuron; DA: dopamine; SN: substantia nigra; 6-OHDA: 6-hydroxydopamine; GDNF: glial cell line-derived neurotrophic factor.
